# The *Drosophila* Importin-α3 Is Required for Nuclear Import of Notch In Vivo and It Displays Synergistic Effects with Notch Receptor on Cell Proliferation

**DOI:** 10.1371/journal.pone.0068247

**Published:** 2013-07-01

**Authors:** Nalani Sachan, Abhinava K. Mishra, Mousumi Mutsuddi, Ashim Mukherjee

**Affiliations:** Department of Molecular and Human Genetics, Banaras Hindu University, Varanasi, India; University of Valencia, Spain

## Abstract

The Notch signaling pathway controls diverse cell-fate specification events throughout development. The versatility of this pathway to influence different aspects of development comes from its multiple levels of regulation. Upon ligand-induced Notch activation, the Notch intracellular domain (Notch-ICD) is released from the membrane and translocates to the nucleus, where it transduces Notch signals by regulating the transcription of downstream target genes. But the exact mechanism of translocation of Notch-ICD into the nucleus is not clear. Here, we implicate Importin-α3 (also known as karyopherin-α3) in the nuclear translocation of Notch-ICD in *Drosophila*. Our present analyses reveal that Importin-α3 can directly bind to Notch-ICD and loss of Importin-α3 function results in cytoplasmic accumulation of the Notch receptor. Using MARCM (Mosaic Analysis with a Repressible Cell Marker) technique, we demonstrate that Importin-α3 is required for nuclear localization of Notch-ICD. These results reveal that the nuclear transport of Notch-ICD is mediated by the canonical Importin-α3/Importin-β transport pathway. In addition, co-expression of both Notch-ICD and Importin-α3 displays synergistic effects on cell proliferation. Taken together, our results suggest that Importin-α3 mediated nuclear import of Notch-ICD may play important role in regulation of Notch signaling.

## Introduction

The Notch pathway is an evolutionarily conserved signaling system which has been shown to play major role in cell fate determination, differentiation, proliferation and apoptotic events as well as self-renewal processes of different tissues [1]–[6]. The same pathway can be deployed in numerous cellular contexts to play varied and critical roles for the development of an organism. The Notch receptor is synthesized as a single polypeptide precursor, which during maturation in the trans-Golgi network is first cleaved by a furin protease into a N-terminal extracellular subunit and a C-terminal transmembrane intracellular subunit [7]. This heterodimeric receptor is then transferred to the cell membrane where it interacts with its ligands, Delta and Serrate in *Drosophila* (Delta and Jagged in vertebrates). Binding of ligands to extracellular domain leads to a metalloprotease-dependent cleavage in the extracellular portion of transmembrane intracellular fragment [8], which is followed by an intramembrane cleavage by the Presenilin-dependent gamma-secretase activity resulting the release of Notch intracellular domain [9]–[12]. The Notch intracellular domain is translocated to the nucleus where it binds to and activates a transcription factor, Suppressor of Hairless in *Drosophila* (CBF1 in vertebrates) [13]–[15]. This complex also recruits Mastermind [16] and other transcriptional coactivators leading to activation of Notch target genes such as the *Enhancer of Split* [*E(spl*)] complex genes [17].

In an effort to identify novel components involved in Notch signaling and its regulation, a yeast two-hybrid screen was carried out using the portion of intracellular domain of Notch receptor as bait and we identified *Drosophila* Importin-α3 as binding partner of Notch. *Drosophila* Importin-α3 protein is known to play major role in nuclear trafficking of different Nuclear Localization Signal (NLS) containing proteins such as Germ Cell-less [18], the large subunit of DNA polymerase α [19], heat shock transcription factor (dHSF) [20], Daxx [21], Naked cuticle (Nkd) [22] etc. Since nuclear transport protein Importin-α3 directly binds to portion of Notch intracellular domain which contains NLS [7], we were prompted to examine if Notch intracellular domain translocates to nucleus using the canonical nuclear transport machinery.

In human, there are seven Importin α family members, whereas *Drosophila* has Importin-α1, Importin-α2 and Importin-α3 coding genes. Among them only Importin-α3 binds to NLS-containing proteins via its Armadillo (Arm) motifs and to Importin-β via its N-terminal Importin-β binding domain (IBB) [23]. Importin-β interacts with nuclear pore complex (NPC) and targets NLS protein/Importin-α3/Importin-β trimeric complex to the nuclear pore for translocation into the nucleus. RanGTP concentration in the nucleus is high and it interacts with Importin-β, resulting in disassembly of the import complex releasing both Importin-α3 and the NLS cargo into the nucleus. Subsequently, Importin-α3 is free and forms a trimeric complex with RanGTP and CAS (Cellular apoptosis susceptibility) proteins. This trimeric complex is then exported to the cytoplasm, recycling Importin-α3 for another round of import. Importin-β is also recycled back to cytoplasm by binding to RanGTP in the nucleus [24].

Our molecular and genetic analyses presented here clearly demonstrate that Importin-α3 plays important role in nuclear transport of Notch-ICD and co-expression of Importin-α3, together with Notch-ICD, displays synergistic effects on signaling activity of the Notch receptor.

## Results and Discussion

### Importin-α3 is an Interacting Partner of Notch

**Figure 1:**
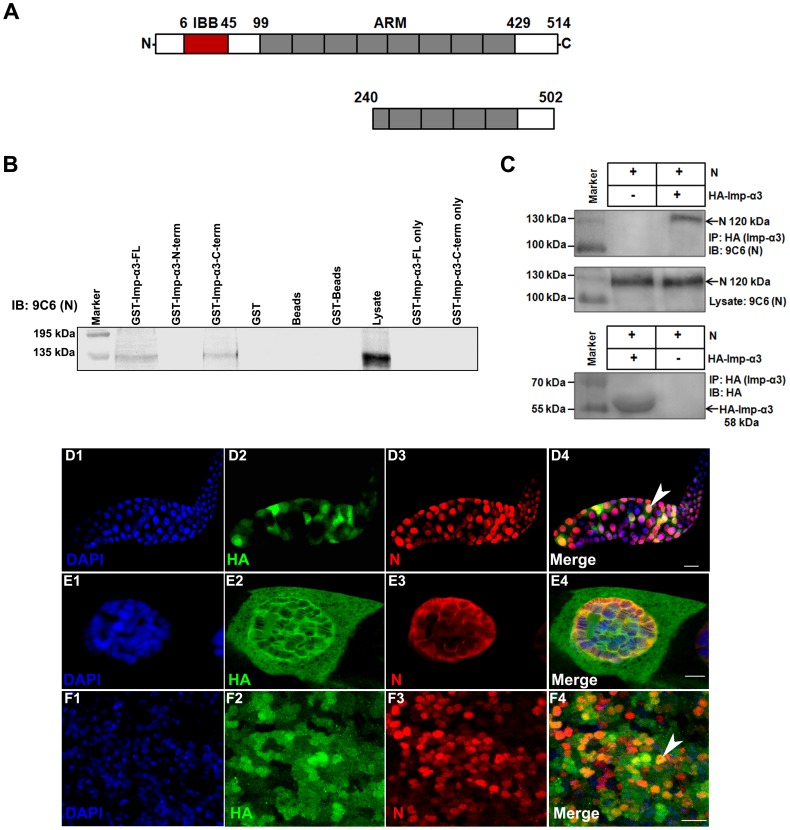
*Drosophila* Notch binds Importin-α3. (A) Schematic representation of the domain organization of Importin-α3. Different domains and boundary residues are marked on top. IBB, Importin β binding domain; ARM, Armadillo repeats [see refs 19, 20]. A region of Importin-α3 (amino acids 240–502) that was sufficient for binding to Notch, based on yeast two-hybrid analysis, is shown below the full-length protein. (B) GST-pulldown assay was performed with lysate of salivary glands in which Notch-ICD was overexpressed using salivary gland specific *GAL4* driver (*sgs-GAL4*) and purified recombinant GST-Importin-α3 full-length (amino acids 1–514), amino-terminal (amino acids 1–224), carboxy-terminal (amino acids 225–514) and other controls as indicated. GST pulled down proteins were analyzed by western blotting with anti-Notch (C17.9C6) antibodies. GST-Importin-α3 full-length and GST-Importin-α3 carboxy-terminus pulled down Notch-ICD. (C) Co-immunoprecipitation of HA-Importin-α3 and Notch-ICD. HA-Importin-α3 and Notch-ICD were co-expressed in larval salivary glands and immunoprecipitated with anti-HA agarose. Immunoprecipitated proteins were analyzed by western blotting with anti-Notch (C17.9C6) antibodies (upper panel) and with anti-HA antibodies (lower panel). Middle panel shows the level of Notch protein in the lysates. (D1–F4) Co-localization of HA-Importin-α3 and Notch-ICD in salivary glands (D1–E4) and eye discs (F1–F4). *UAS-HA-imp-α3* and *UAS-Notch-ICD* were expressed under the control of the *ey-GAL4* driver. Images in D4, E4, and F4 are merges of those in D1–D3, E1–E3, and F1–F3, respectively. Images in E1–E4 are high magnification images of a single cell from salivary glands shown in D1–D4. Co-expression of *HA-Importin-α3* and *Notch-ICD* shows their co-localization in cell nuclei (arrowheads). Scale bars, 100 µm (D1–D4), 10 µm (E1–F4).

In a yeast two-hybrid screen, we identified Importin-α3 as an interacting partner of Notch. In the same screen, multiple positive clones of a well established binding partner of Notch-ICD, Suppressor of Hairless, were also identified, which validates our approach. The yeast two-hybrid screen of 6×10^6^ cDNAs from a *Drosophila* 0–24 h embryonic library was carried out using amino terminus of Notch intracellular domain (amino acids 1765–1895) as bait. Twenty one positive clones (His^+^) were isolated and found to encode overlapping *imp-α3* cDNAs. Sequence analysis of these clones revealed that the carboxy-terminal part of Importin-α3 (amino acids 240–502) is necessary and sufficient for binding Notch (Figure 1A). This particular domain of Importin-α3 was shown earlier to interact with NLS containing proteins [25].

GST-pull down experiments using purified GST-Importin-α3 confirmed the interaction between Notch and Importin-α3. Different GST-Importin-α3 fusion proteins (full-length 1–514, amino terminus 1–224 and carboxy terminus 225–514) were expressed in bacteria and fusion products were isolated on Glutathione Sepharose beads. After extensive washing, the beads were incubated with extracts from third instar larval salivary glands in which Notch-ICD was overexpressed using *ey-GAL4* driver. Deletion analysis of Importin-α3 protein demonstrated that carboxy-terminus portion of Importin-α3 is required for binding to Notch-ICD (Figure 1B). Furthermore, co-immunoprecipitation experiment was carried out in which Notch-ICD was immunoprecipitated with HA-Importin-α3 from larval salivary glands when both proteins were co-expressed (Figure 1C). Taken together, these results suggest that the Importin-α3 directly interacts with Notch and that the Importin-α3 binds with Notch through its C-terminus that is known to bind with NLS-containing proteins. To further analyze interactions between Importin-α3 and Notch, we investigated the subcellular localization of these proteins when *UAS-HA-imp-α3* and *UAS-Notch-ICD* were co-expressed in larval salivary glands and eye imaginal discs using *ey-GAL4* driver. Immunocytochemical analysis revealed that Importin-α3 and Notch-ICD indeed co-localized in cell nuclei (Figure 1D1–1F4).

### Genetic Interactions between *imp-α3 and Notch* Pathway Components

**Figure 2:**
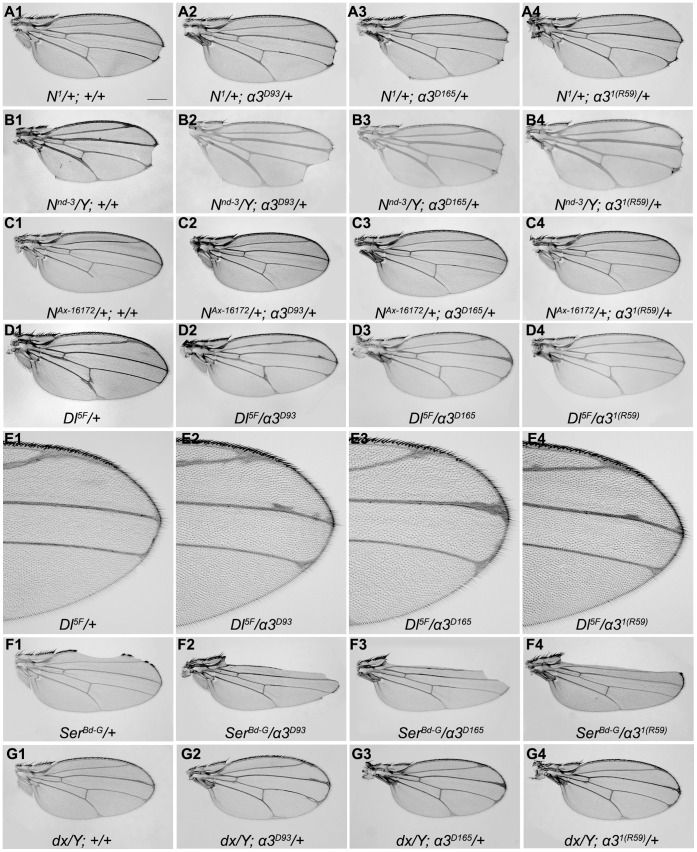
Genetic interactions of *imp-α3* with Notch pathway components. (A1–G4) Representative wings from individuals with indicated genotypes. Wings from *N^1^* heterozygotes (A1) show wing notching phenotype which was enhanced in transheterozygous combination with different alleles of *imp-α3* (A2–A4). Wing notching phenotype of *N^nd-3^* hemizygotes (B1) was enhanced in combination with *imp-α3* alleles (B2–B4). Wing vein phenotype involving a shortened longitudinal vein V of *N^Ax-16172^* mutation (C1) was rescued in transheterozygous combination with different alleles of *imp-α3* (C2–C4). Wing vein thickening phenotype of *Dl* (D1 and E1) was enhanced in combination with *imp-α3* alleles (D2–D4 and E2–E4). Images in E1–E4 are high-magnification images of the distal ends of wings shown in D1–D4, respectively. Wing nicking phenotype of *Ser^Bd-G^* (F1) was enhanced in transheterozygous combination with different alleles of *imp-α3* (F2–F4). Wing phenotype of *dx* hemizygotes (G1) with *imp-α3* mutants showed enhanced phenotype that consist of extra vein material at the distal ends of wing veins (G2–G4). n = 100 wings for each genotype. The expressivity and penetrance of the phenotype for each genotype were 100% except for *N^1^* heterozygotes (A1) and *N^1^* heterozygotes with different alleles of *imp-α3* (A2–A4) in which penetrance of the phenotype was 45%. Scale bar, 250 µm.

To address functional implications of the physical interaction between the Importin-α3 and Notch proteins, we investigated whether mutations in *imp-α3* and *Notch* or other components involved in Notch signaling pathway display genetic interactions in transheterozygous combinations. We used two independent loss-of-function *imp-α3* alleles: *imp α3^D93^* and *imp α3^D165^* and one hypomorphic allele, *imp α3^1(R59)^* [26]. A transheterozygous combination of *Notch* null allele, *N^1^*or a hemizygous *Notch* hypomorphic allele, *N^nd-3^* and any one of the three *imp-α3* alleles resulted in enhancement of wing nicking phenotype, indicating further reduction of the Notch function (Figure 2A1–2B4). On the contrary when we used gain-of-function *Notch* allele, the *Abruptex* mutation (*N^Ax-16172^*) which displays a shortened longitudinal vein V (L5), we noticed an extension of L5 vein upto the wing margin in transheterozygous combination with *imp-α3* mutations (Figure 2C1–2C4). The wing vein thickening phenotype of *Delta* (*Dl)* and the wing notching phenotype of the dominant negative mutation of *Serrate* (*Ser^Bd-G^*) were also enhanced by reducing the dose of *imp-α3* (Figure 2D1–2F4). A transheterozygous combination of *dx*, which is a cytoplasmic modulator of Notch activity, and *imp-α3* alleles resulted in normal wings (data not shown) whereas *dx* in hemizygous combination with *imp-α3* alleles showed wing phenotype that consist of extra vein material at the distal ends of wing veins (Figure 2G1–2G4). These observations confirm a functional relationship between *imp-α3* and *Notch* consistent with their molecular interactions.

### *imp-α3* Mutant Cells have Elevated Notch Protein Levels

**Figure 3:**
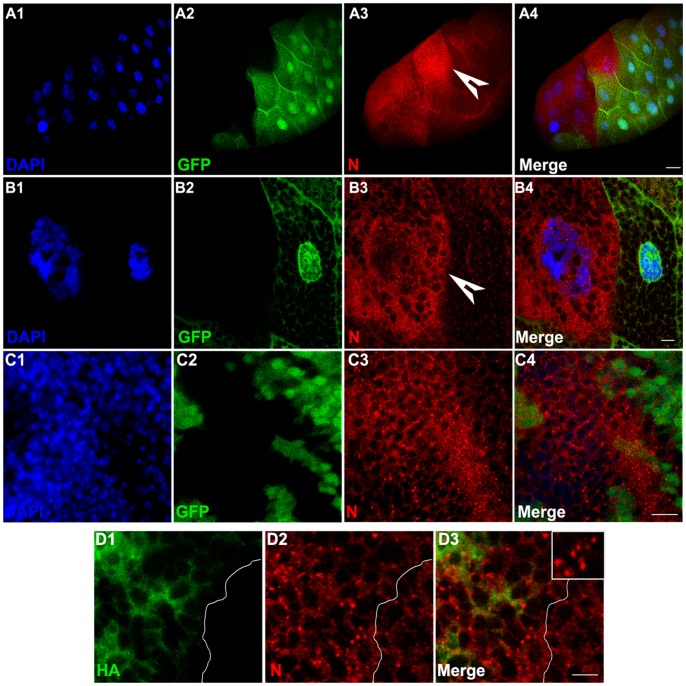
Loss-of-function and gain-of-function effects of *imp-α3* on localization of endogenous Notch protein. (A1–C4) *imp-α3* mutant cells have elevated Notch protein levels. Levels of Notch (N) protein in third instar larval salivary glands (A1–B4) and eye-antennal discs (C1–C4) that contain *imp-α3* mutant clones marked by the absence of green fluorescent protein (GFP). Images in A4, B4, and C4 are merges of those in A1–A3, B1–B3, and C1–C3, respectively. High magnification of *imp-α3* clones in A1–A4 are shown in B1–B4. Note the increased levels of Notch in *imp-α3* mutant cells (arrowheads). Scale bars, 50 µm (A1–A4), 10 µm (B1–C4). (D1–D3) Ectopic expression of Importin-α3 results in the formation of cytoplasmic aggregates of endogenous Notch protein. *UAS-HA-imp-α3* transgene was expressed under the control of *en-GAL4* driver, which is expressed in posterior compartment cells of wing discs. Note that more number of Notch aggregates in cytoplasm of posterior compartment cells compare to anterior compartment cells in wing disc. Image in D3 is merge of those in D1 and D2. Inset in D3 shows higher magnification image of a single cell from posterior compartment showing many Notch aggregates. Scale bar, 5 µm.

To further analyze the molecular implications of Importin-α3 and Notch interaction *in vivo*, we examined the effects of *imp-α3* loss-of-function on the endogenous Notch protein. We generated *imp-α3* loss-of-function clones in two different larval tissues, eye-antennal imaginal discs and salivary glands, using *imp α3^D93^* null mutant [26] and the FLP/FRT system [27]. The FLP activity that is under the control of *eyeless* promoter (*ey-FLP*) was used to induce somatic recombination events in eye discs, while *hsFLP* was used to generate somatic clones in salivary glands. Salivary glands begin to develop at 4.5 hours of development and complete by 10 hours of embryonic development. Thus, to generate somatic clones in salivary glands, 4–8 hours old embryos were subjected to a single heat shock (37°C for 45 min). The *imp-α3* mutant clones in either tissues were identified by the absence of GFP expression. Notch protein levels in the cytoplasm were strongly elevated in *imp-α3* mutant cells compared with the surrounding wild-type cells in both salivary glands and eye-antennal discs (Figure 3A1–3C4). These elevated Notch protein levels in *imp-α3* mutant cells may be due to the lack of nuclear transport of Notch-ICD in the absence of Importin-α3.

### Gain-of-function Effect of *imp-α3* on Notch Protein

In parallel with the *imp-α3* loss-of-function analysis, we also determined the gain-of-function effect of *imp-α3* by monitoring the effects of its ectopic expression on Notch localization. We examined the distribution of endogenous Notch protein in wing imaginal discs in which *imp-α3* expression was driven by *en-GAL4* driver. Expression of *UAS-HA-imp-α3* with *en-GAL4* driver resulted in cytoplasmic aggregates of Notch protein in posterior compartment cells of wing discs (Figure 3D1–3D3, also see Figure S1F1–S1F3). Since *en-GAL4* is a posterior compartment specific driver, cells from anterior compartment serve as an internal control (Figure 3D1–3D3, and S1E1–S1E3). This effect is specific for *imp-α3* since when the other two importins, *imp-α1* and *imp-α2*, were overexpressed in wing discs using *en-GAL4* driver, cytoplasmic aggregates of Notch were never observed (see Figure S1A1–S1D3). Thus, this effect on Notch accumulation is a specific consequence of *imp-α3* expression. Our analysis does not exclude the possibility that these aggregates may be some kind of vesicular structures which remains to be determined.

### Importin-α3 is Required for Notch Nuclear Localization

**Figure 4:**
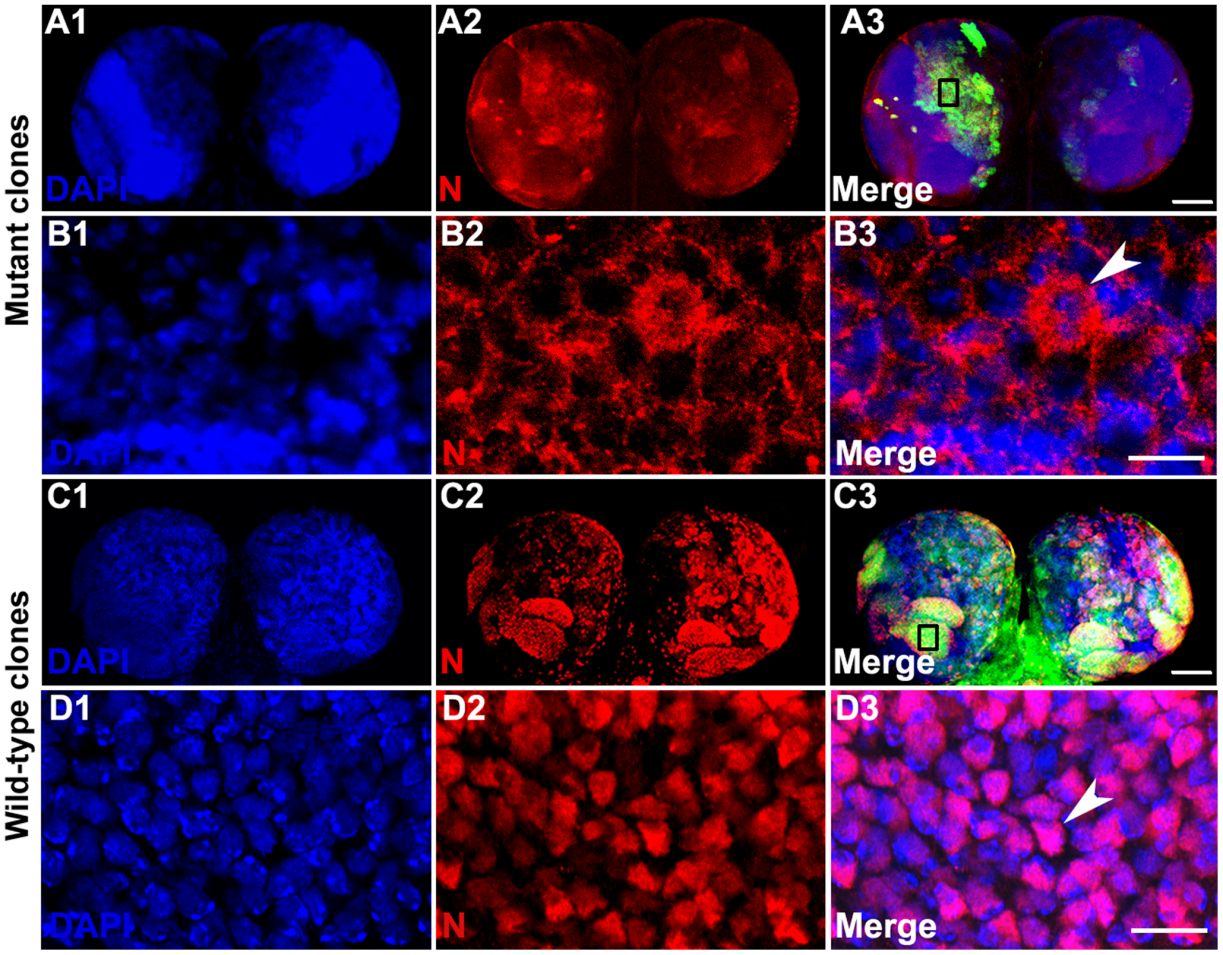
Loss of ***imp-α3*** blocks the nuclear import of Notch-ICD. (A1–D3) MARCM-derived *imp-α3* mutant clones (A1–B3) and wild-type clones (C1–D3) in larval brain marked with green fluorescent protein (GFP). Images in A3, B3, C3, and D3 are merges of those in A1–A2, B1–B2, C1–C2, and D1–D2, respectively. High magnification of part (marked with open rectangle in A3 and C3) of GFP-marked *imp-α3* clones in A1–A3 are shown in B1–B3 and GFP-marked wild-type clones in C1–C3 are shown in D1–D3. Note the cytoplasmic localization of Notch-ICD in *imp-α3* mutant cells (arrowhead in B3), which is readily detectable in the nucleus in wild-type clonal cells as shown in D3 (arrowhead). Scale bars, 100 µm (A1–A3 and C1–C3), 10 µm (B1–B3 and D1–D3).

Endogenous Notch-ICD is not easily detectable in nucleus by immunostaining using antibody specific for intracellular domain of Notch, since very little amount of the cleaved product is translocated to nucleus for carrying out its downstream function [28], [29]. Recently it has been reported that endogenous Notch-ICD is detectable in the nucleus of pIIa cells derived by asymmetric division of Sensory organ precursor cells (SOPs) [30]. It was not straightforward to test whether transport of endogenous Notch-ICD was inhibited in the absence of Importin-α3. When Notch-ICD is overexpressed, it is readily detectable in the nucleus [31]–[33] (also see Figure 1D1–1F4). To determine the role of Importin-α3 in nuclear localization of Notch we overexpressed *Notch-ICD* in *imp-α3* mutant clonal cells using the MARCM technique [34]. Although Notch-ICD was under the control of *UAS* and the *Tub-GAL4* driver was used to globally drive *Notch-ICD* expression, the presence of GAL80 inhibited GAL4-induced expression of *Notch-ICD* in all cells except in those cells in which GAL80 was eliminated due to FLP-FRT mediated somatic recombination events. At the same time *imp-α3* gene function was also eliminated in same cells. As a result, these *imp-α3* mutant clonal cells, which were also marked with GFP, overexpressed *Notch-ICD* under *Tub-GAL4* driver. In parallel, MARCM analysis of wild-type clones without *imp-α3* mutation was also carried out and subcellular localization of Notch protein was determined. In GFP-marked *imp-α3* mutant clonal cells, Notch-ICD was completely excluded from the nucleus (Figure 4A1–4B3), whereas Notch-ICD was readily detectable in the nucleus of wild-type clonal cells without *imp-α3* mutation (Figure 4C1–4D3).

### Importin-α3 Displays Synergistic Effects with Notch Signals on Cell Proliferation

**Figure 5:**
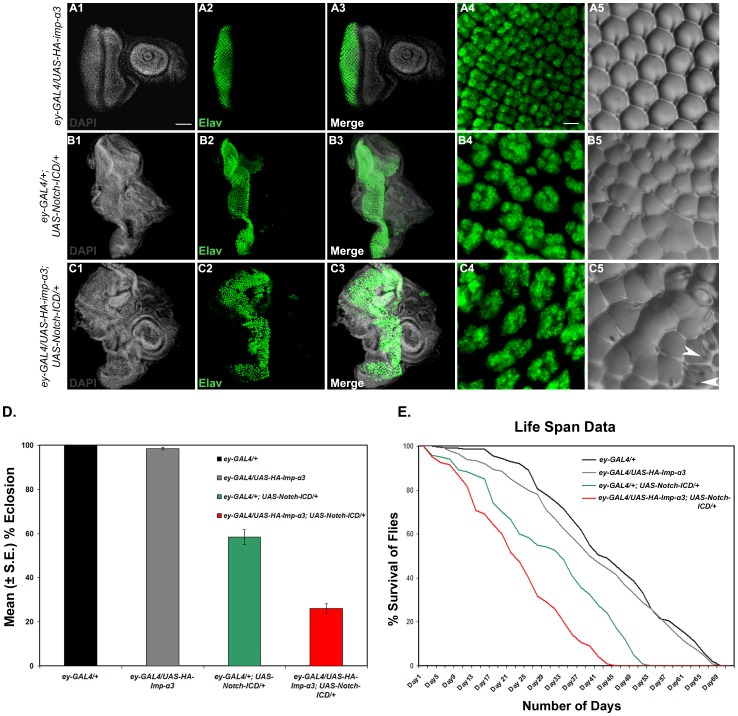
Importin-α3 displays synergistic effect with activated Notch on signaling activity of the Notch receptor. (A1–C5) Eye-antennal discs in which *UAS-HA-imp-α3* was overexpressed by *ey-GAL4* driver (A1–A4), eye-antennal discs in which *UAS-Notch-ICD* was overexpressed using *ey-GAL4* driver (B1–B4), and eye-antennal discs from individuals in which both *UAS-Notch-ICD* and *UAS-HA-imp-α3* were overexpressed by *ey-GAL4* strain (C1–C4) showing Elav expression. Note that Elav staining of larval eye discs in which *Notch-ICD* and *imp-α3* were both overexpressed showed enhanced defects in ommatidial spacing and misrotated ommatidia. Images in A3, B3, and C3 are merges of those in A1 and A2, B1 and B2, and C1 and C2, respectively. Images in A4, B4, and C4 are high magnification images of Elav expressing cells shown in A2, B2, and C2, respectively. Scale bars for A1–A3, B1–B3, and C1–C3, 50 µm and for A4, B4, and C4, 5 µm. (A5, B5, and C5) Nail polish imprints of adult eyes of genotypes as in A1–A4, B1–B4, and C1–C4, respectively. Note the co-expression of *Notch-ICD* and *imp-α3* results in a considerable enhancement of the adult eye phenotype with more frequent fusion of ommatidia and appearance of abnormally sized ommatidia with extra bristles (arrowheads in C5). (D) Histograms show mean percentage of flies eclosed from pupae of different genotypes: *ey-GAL4/+* (Black), *ey-GAL4/UAS-HA-imp-α3* (Grey), *ey-GAL4/+; UAS-Notch-ICD/+* (Green), and ey*-GAL4/UAS-HA-imp-α3; UAS-Notch-ICD/+* (Red). Note that 58% *ey-GAL4* driven Notch-ICD overexpressing pupae emerged as adult flies and this was reduced to 23% in which both *Notch-ICD* and *imp-α3* were overexpressed. The bars represent mean (± S.E.) of 9 replicates (n = 50 in each replicate; total n for each genotype = 450). (E) The survival curves of different genotypes: *ey-GAL4/+* (Black), *ey-GAL4/UAS-HA-imp-α3* (Grey), *ey-GAL4/+; UAS-Notch-ICD/+* (Green), and ey*-GAL4/UAS-HA-imp-α3; UAS-Notch-ICD/+* (Red). Note that both *HA-imp-α3* and *Notch-ICD* expressing flies showed significantly reduced life span as compared to only *HA-imp-α3* or *Notch-ICD* overexpressing flies.

Earlier studies have shown that overexpression of activated form of Notch (Notch-ICD) in eye discs results in roughening of the eye with fused or missing ommatidia and bristle irregularities [33]. We have also observed that overexpression of Notch-ICD using *ey-GAL4* driver exhibits rough eye phenotype with fused or abnormally sized ommatidia together with extra and missing bristles (Figure 5B5). Immunostaining of Elav in eye-discs revealed that overexpression of Notch-ICD results in fusion of ommatidia and defective ommatidial spacing (Figure 5B1–5B4). Interestingly, co-expression of Notch-ICD and Importin-α3 using the same *ey-GAL4* driver results in a considerable enhancement of the adult eye phenotype with more frequent fusion of ommatidia and appearance of abnormally sized ommatidia with extra bristles (Figure 5C5). Similarly, Elav staining of larval eye discs in which both Notch-ICD and Importin-α3 were overexpressed also showed enhanced defects in ommatidial spacing and misrotated ommatidia (Figure 5C1–5C4). We have also noticed that larval eye discs as well as wing discs, in which both Notch-ICD and Importin-α3 were overexpressed, are considerably larger than only Notch-ICD overexpressing eye or wing discs and these discs are thicker, wrinkled and highly distorted as compared to only Notch-ICD overexpressing discs (Figure 5A1, 5B1, 5C1, and S2). These results reveal a synergistic effect between Importin-α3 and Notch-ICD on cell proliferation in eye and wing discs.

We have observed that 58% *ey-GAL4* driven Notch-ICD overexpressing pupae (n = 450) emerged as adult flies and this was reduced to 23% in which both Notch-ICD and Importin-α3 were overexpressed (n = 450) using the same *ey-GAL4* driver (Figure 5D). Moreover, flies that survived in which both Notch-ICD and Importin-α3 were overexpressed have significantly reduced life span as compare to only Notch-ICD overexpressing flies (Figure 5E).

It has previously been shown that mammalian Notch1-ICD must accumulate in the nucleus to induce neoplastic transformation of baby rat kidney cells (RKE) [35]. Another report has described that downregulation of importins α3, α5, α7 and Importin β strongly inhibits HeLa cell proliferation and on the basis of these findings it was proposed that import pathways of various substrates that are essential for cell proliferation were blocked, resulting in proliferation inhibition [36]. Interestingly, our data also suggest that overexpression of Importin-α3 along with Notch-ICD displays a synergistic effect between Importin-α3 and Notch activation on cell proliferation in the eye and wing imaginal discs which is likely to be caused by stronger activation of Notch signals due to the greater import of Notch-ICD into the nucleus by overexpressed Importin-α3.

Our results establish that the nuclear transport of Notch-ICD is mediated by the canonical Importin-α3/Importin-β transport pathway and co-expression of both Notch-ICD and Importin-α3 displays synergistic effects on cell proliferation. Earlier a genome-wide loss-of-function analysis by transgenic RNAi in *Drosophila* has been carried out to study the Notch signaling pathway during external sensory organ development and many novel components including nuclear import pathway components and nuclear pore components have been identified as Notch regulators [37]. Although it has been documented through cell culture based experiments that Importins α3, α4 and α7 mediate nuclear import of Notch-ICD in mouse myoblast and human HeLa cells [38], our present report is the first *in vivo* study showing role of Importin-α3 in nuclear import of Notch-ICD in *Drosophila* and its synergistic effects with Notch signals on cell proliferation.

Notch signaling is known to affect a broad spectrum of cell-fate decisions throughout development. To allow the Notch signal to be deployed in numerous cellular contexts, many different mechanisms have evolved to regulate the level, duration, and spatial distribution of Notch activity [2], [4], [39]. It is known that the transport of proteins and RNAs in and out of nucleus plays important role in the regulation of gene expression during every stage of development and tissue differentiation [40]. Nuclear import of Notch-ICD may play important role in regulation of Notch signaling activity and it remains possible that this mode of regulation may be involved in many other signaling pathways.

## Materials and Methods

### Yeast Two-hybrid

A 393 bp *Drosophila* Notch cDNA (accession number M11664) fragment which encodes amino acids 1765–1895 containing NLS was amplified by polymerase chain reaction (PCR) and cloned in frame with the sequence encoding the LexA DNA-binding domain of bait vector. This construct was used as bait to screen oligo(dT)-primed *D. melanogaster* 0–24 h embryo cDNA libraries cloned in pGAD prey vectors containing GAL4 activation domains. A yeast two-hybrid screen was carried out as described previously [39]. Finally, all positive pGAD plasmids from His+ colonies were isolated and sequenced to identify interactors.

### GST Pulldown, Immunoprecipitation and Immunoblotting

For GST-pulldown, DNA fragments coding for full-length Importin-α3 (amino acids 1–514), N-terminal Importin-α3 (amino acids 1–224), and C-terminal Importin-α3 (amino acids 225–514) were cloned into pGEX-4T-1 vector (Amersham). The following forward and reverse primers were used for PCR amplification of different fragments of *imp-α3* (GenBank accession number AY069430):

Full-length *imp-α3*- 5′CGCAGGAATTCATGACGTCTATGGAGCAAAATC3′and 5′GCGAGGCGGCCGCTTAAAAGTTAAATGAGTTC3′,

N-terminal *imp-α3*- 5′CGCAGGAATTCATGACGTCTATGGAGCAAAATC3′ and 5′GCGAGGCGGCCGCTTAGCGGCACAAATTCAC3′, and C-terminal *imp-α3*-.

5′CGCAGGAATTCAACAAGGATCCGGCTC3′ and.

5′GCGAGGCGGCCGCTTAAAAGTTAAATGAGTTC 3′ primers.

Intact reading frames for all constructs were verified by DNA sequence analysis. GST and GST fusion proteins were expressed in *E. coli* BL21 cells at 37°C with 2 mM isopropyl-1-thio-β-D-galactopyranoside (IPTG) induction. Bacteria were lysed in solution of Cell Lytic™ express tablet (Sigma) with complete protease inhibitor (Roche). Glutathione Sepharose (GE Healthcare Bio-Sciences) beads were washed in cold phosphate buffered saline (PBS), 3 times 30 minutes each. 50% slurry was made in PBS.

Intracellular domain of Notch was overexpressed in salivary glands by salivary gland specific GAL4 driver (*sgs–GAL4)* and third instar larval salivary glands were dissected and washed in PBS, then lysed in lysis buffer (50 mM Tris pH8.0, 0.1% TritonX-100, 10% Glycerol, 200 µg/ml lysozyme and 1 mM PMSF) for 3 hrs at 4°C.The supernatant was collected after centrifugation for 20 min at 12,000 rpm.

Glutathione Sepharose beads alone or incubated with GST fusion proteins mixed with salivary gland lysate and rotated for 3 hrs at 4°C followed by three times washing with PBST (1X PBS, 1% Triton-X-100), 15 min each. Beads were boiled in 2X Laemmli buffer for 5 min and samples were loaded in 12% denaturing gel with Spectra multicolor broad range protein ladder used as a marker (Fermentas). Proteins were separated on non-reducing SDS-PAGE (without β-mercaptoethanol) and transferred onto PVDF membrane (Bio-Rad). Blot was probed with mouse anti-Notch C17.9C6 in 1∶3000 dilution (Developmental Studies Hybridoma Bank) and secondary antibody goat anti-mouse IgG-AP conjugate in 1∶2000 dilution (Molecular Probes) in blocking solution (4% skimmed milk in TBST-50 mM Tris, pH 7.5, 150 mM Nacl, 0.1% Tween-20**)**. Then after washing in TBST thrice, colour was detected by Sigma FAST™ BCIP/NBT (Sigma).

Immunoprecipitation from larval salivary glands was carried out as described previously [39]. HA-Importin-α3 and Notch-ICD proteins were expressed in larval salivary glands under the control of *sgs-GAL4* driver and immunoprecipitated with anti-HA affinity beads (Sigma). For detection of Notch, we used monoclonal mouse anti-Notch (C17.9C6) antibody and for detection of HA, we used mouse anti-HA antibody at 1∶1000 dilution (Sigma).

### Drosophila Genetics

All fly stocks were maintained on standard cornmeal/yeast/molasses/agar medium at 25°C. The *imp α3^1(R59)^/TM6C*, *imp α3^D93^/TM6B, imp α3^D165^/TM3*, FRT82B *imp α3^D93^/TM6B*, UASp *imp α1^(T 2–1)^/CyO*, UASp *imp α2^(T2 1-2)^/TM6B*, UASp *imp α3^(T3 2-1)^/CyO* stocks were obtained from Robert J. Fleming. We used the following alleles of Notch pathway components (kindly provided by S. Artavanis-Tsakonas) for genetic interaction studies: *N^1^*, *N^nd-3^*, *N^Ax-16172^*, *Dl^5F^*, *Ser^Bd-G^*, *dx^152^*. To generate somatic clones using the FLP/FRT system, the following stocks were used: *y w hsFLP*; *FRT82B Ubi–GFP*/*TM6B* and *y w ey–FLP*; *FRT82B Ubi–GFP*/*TM6B* which were obtained from Bloomington Drosophila Stock Center (Bloomington, IN). To generate somatic clones in salivary glands, 4–8 hours old embryos were subjected to a single heat shock (37°C for 45 min). For generation of the *P[UAS–HA–imp-α3]*, a full-length *imp-α3* cDNA with a HA tag at the amino-terminus was cloned in the pUAST vector. This construct was introduced into *w^1118^* embryos by germline transformation according to the standard procedures. Multiple independent insertions were obtained. The UAS constructs were expressed under the control of *ey–GAL4*, *en–GAL4, sgs-GAL4* and *ap-GAL4* drivers. To co-express Importin-α3 and Notch-ICD, *w; UAS–HA–imp-α3/CyO; UAS-Notch-ICD/TM6B* stock was generated by appropriate genetic crosses.

### MARCM Clonal Analysis

The MARCM system was used to generate GFP-marked *imp-α3* mutant clones overexpressing *Notch-ICD*. First *UAS-Notch-ICD*; *FRT82B imp α3^D93^/TM6B* stock was generated by appropriate genetic crosses. To generate GFP-marked clones, females of *UAS-Notch-ICD*; *FRT82B imp α3^D93^/TM6B* were crossed to male *y w hsFLP Tub-GAL4 UAS-GFP; FRT82B Tub-GAL80* flies. In parallel, a control experiment was carried out in which females of *UAS-Notch-ICD*; *FRT82B/TM6B* were crossed to male flies of *y w hsFLP Tub-GAL4 UAS-GFP; FRT82B Tub-GAL80* genotype. Heat shock was given at 37°C for 45 min at 24 hrs AEL and third instar female larvae were analyzed for GFP-marked clones.

### Immunocytochemistry and Confocal Microscopy

*Drosophila* third instar larval imaginal discs, brain and salivary glands were dissected in cold PBS. Immunostaining of these tissues was performed as described previously [39]. Primary antibodies, mouse anti-Notch (C17.9C6) at 1∶300 dilution, Rat anti-Elav at 1∶200 (Developmental Studies Hybridoma Bank), Rabbit anti-HA at 1∶100 (Sigma) and secondary antibodies, goat anti-mouse antibodies Alexa Flour 555 at a 1∶200 dilution (Invitrogen), goat-anti-rat antibodies conjugated to FITC at a 1∶200 dilution (Sigma), goat-anti-rabbit antibodies conjugated to FITC at a 1∶200 dilution (Jackson ImmunoResearch Laboratories, West Grove, PA) were used for immunostaining. After washing secondary antibodies, DAPI (4′, 6-diamidino-2-phenylindole dihydrochloride) was used to detect the nucleus and washed twice in PBS for 10 min each. Then larval tissues were mounted in FluoroGuard Antifade Reagent (Bio-Rad) and images were captured with a Zeiss LSM510 Meta laser confocal microscope.

## Supporting Information

Figure S1 **Overexpression of Importin-α3 specifically results in the formation of cytoplasmic aggregates of endogenous Notch protein.** (A1–F3) *UAS-imp α1, UAS-imp α2, and UAS-imp α3* transgenes were expressed under the control of *en-GAL4* driver, which is expressed in posterior compartment cells of wing discs. Localization of endogenous Notch protein in anterior compartment (A1–A3) and posterior compartment (B1–B3) of a wing disc in which *UAS-imp α1* expression was driven by *en-GAL4*. Similarly, localization of Notch protein in anterior compartment (C1–C3) and posterior compartment (D1–D3) of a wing disc in which *UAS-imp α2* was overexpressed and distribution of Notch protein in anterior compartment (E1–E3) and posterior compartment (F1–F3) of a wing disc in which *UAS-imp α3* was overexpressed. Note that there is no difference in Notch localization in anterior and posterior compartment in case of *UAS-imp α1* and *UAS-imp α2* overexpression (A1–D3) while presence of more number of Notch aggregates in cytoplasm of posterior compartment cells (F1–F3) compare to anterior compartment cells (E1–E3) in *UAS-imp α3* overexpressed wing disc. Images in A3, B3, C3, D3, E3, and F3 are merges of those in A1 and A2, B1 and B2, C1 and C2, D1 and D2, E1 and E2, and F1 and F2, respectively. Insets in A3, B3, C3, D3, E3, and F3 show higher magnification images of a single cell in the corresponding disc. Scale bar, 10 µm.
(TIF)

Figure S2 **Importin-α3 displays synergistic effect with activated Notch on cell proliferation in wing disc.** (A-C) Wing imaginal discs of different genotypes: *ap-GAL4/UAS-HA-imp-α3* (A), *ap-GAL4/+; UAS-Notch-ICD/+* (B), and *ap-GAL4/UAS-HA-imp-α3; UAS-Notch-ICD/+* (C). Note that wing imaginal disc in which both *Notch-ICD* and *HA-imp-α3* were overexpressed (C) is considerably larger than only *HA-imp-α3* (A) or *Notch-ICD* (B) overexpressing wing disc. Scale bar, 100 µm.
(TIF)
