# Supplementary figures and images for: The Drosophila Importin-α3 Is Required for Nuclear Import of Notch In Vivo and It Displays Synergistic Effects with Notch Receptor on Cell Proliferation
Source: PLoS One. 2013 Jul 1;8(7):e68247. doi: 10.1371/journal.pone.0068247 (PMC3698139; doi:10.1371/journal.pone.0068247)

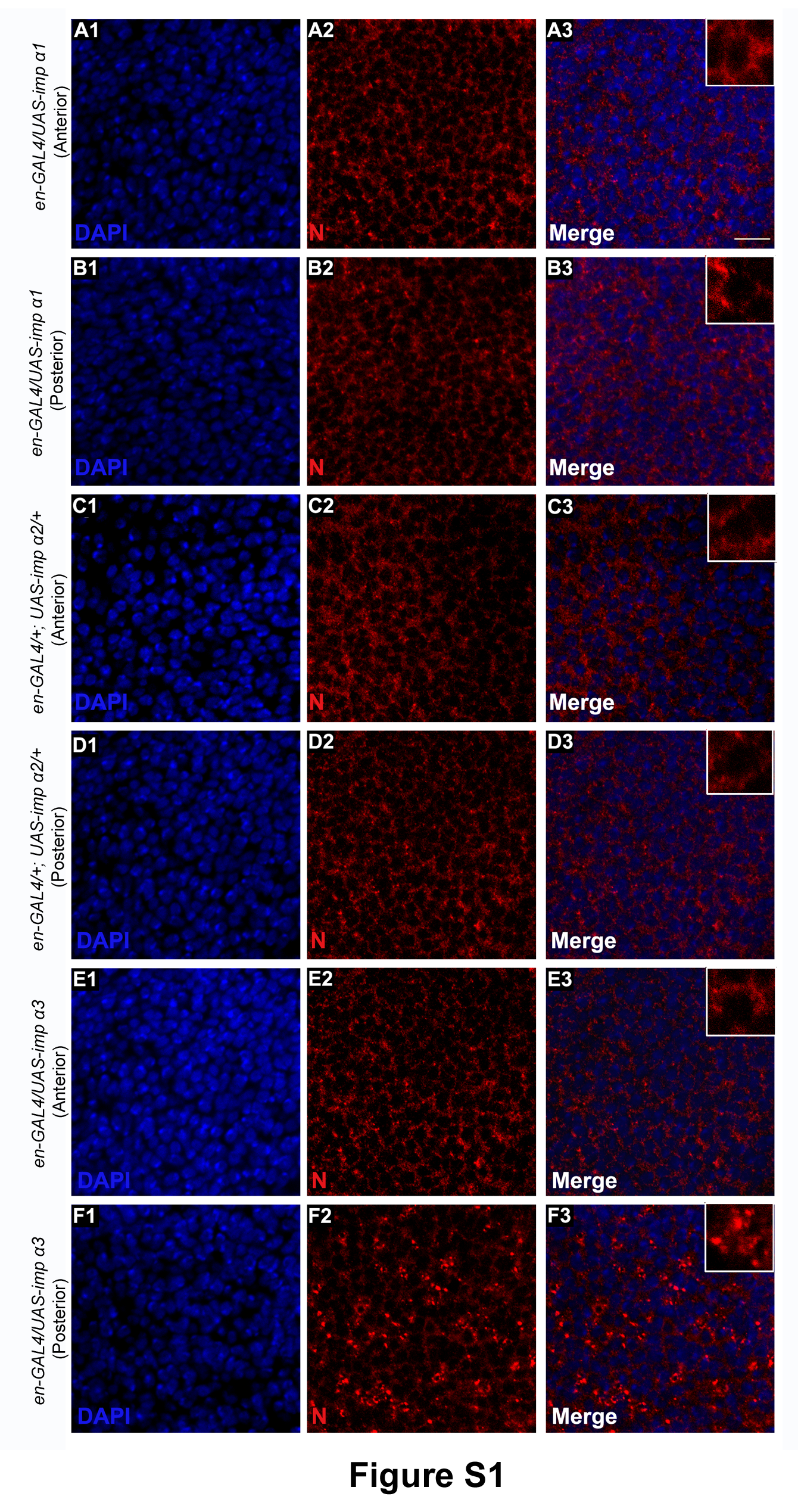

Supplement: Figure S1 — Overexpression of Importin-α3 specifically results in the formation of cytoplasmic aggregates of endogenous Notch protein. (A1–F3) UAS-imp α1, UAS-imp α2, and UAS-imp α3 transgenes were expressed under the control of en-GAL4 driver, which is expressed in posterior compartment cells of wing discs. Localization of endogenous Notch protein in anterior compartment (A1–A3) and posterior compartment (B1–B3) of a wing disc in which UAS-imp α1 expression was driven by en-GAL4. Similarly, localization of Notch protein in anterior compartment (C1–C3) and posterior compartment (D1–D3) of a wing disc in which UAS-imp α2 was overexpressed and distribution of Notch protein in anterior compartment (E1–E3) and posterior compartment (F1–F3) of a wing disc in which UAS-imp α3 was overexpressed. Note that there is no difference in Notch localization in anterior and posterior compartment in case of UAS-imp α1 and UAS-imp α2 overexpression (A1–D3) while presence of more number of Notch aggregates in cytoplasm of posterior compartment cells (F1–F3) compare to anterior compartment cells (E1–E3) in UAS-imp α3 overexpressed wing disc. Images in A3, B3, C3, D3, E3, and F3 are merges of those in A1 and A2, B1 and B2, C1 and C2, D1 and D2, E1 and E2, and F1 and F2, respectively. Insets in A3, B3, C3, D3, E3, and F3 show higher magnification images of a single cell in the corresponding disc. Scale bar, 10 µm. (TIF) [file pone.0068247.s001.tif]

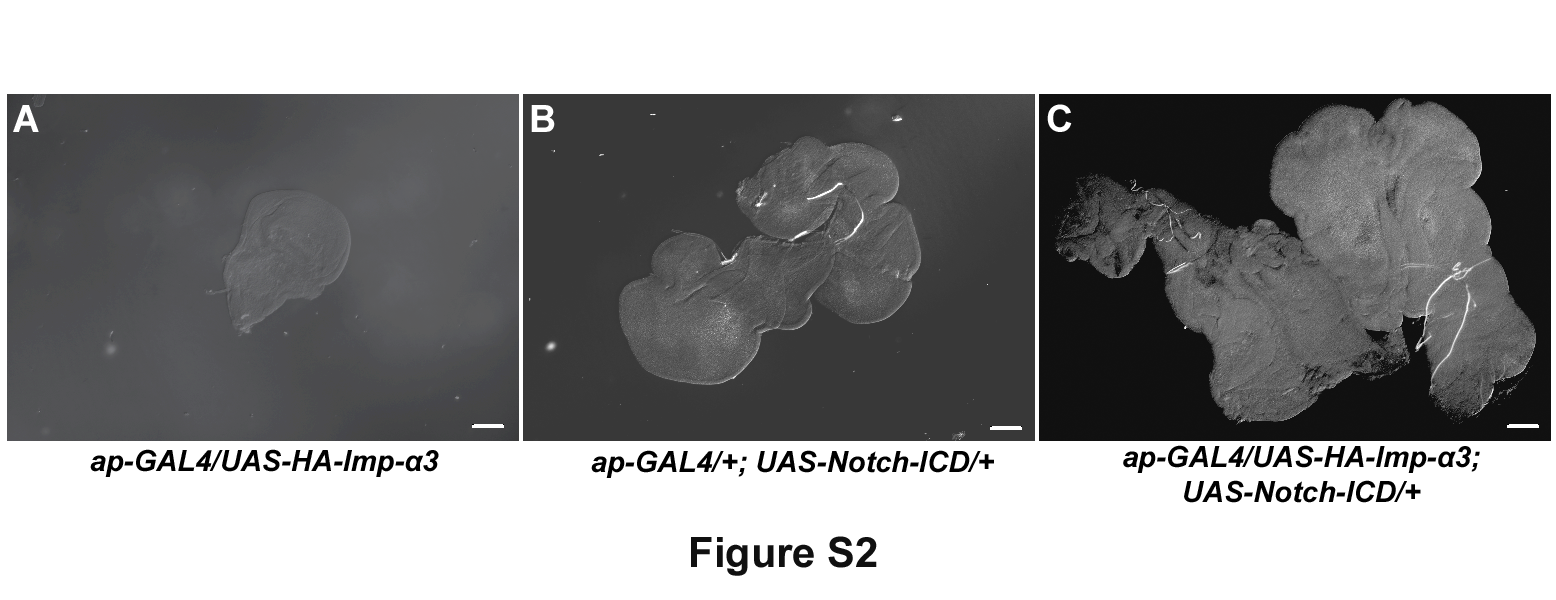

Supplement: Figure S2 — Importin-α3 displays synergistic effect with activated Notch on cell proliferation in wing disc. (A-C) Wing imaginal discs of different genotypes: ap-GAL4/UAS-HA-imp-α3 (A), ap-GAL4/+; UAS-Notch-ICD/+ (B), and ap-GAL4/UAS-HA-imp-α3; UAS-Notch-ICD/+ (C). Note that wing imaginal disc in which both Notch-ICD and HA-imp-α3 were overexpressed (C) is considerably larger than only HA-imp-α3 (A) or Notch-ICD (B) overexpressing wing disc. Scale bar, 100 µm. (TIF) [file pone.0068247.s002.tif]
